# Estimating relative survival among people registered with cancer in England and Wales

**DOI:** 10.1038/sj.bjc.6690005

**Published:** 1999-09-24

**Authors:** G K Reeves, V Beral, D Bull, M Quinn

**Affiliations:** Imperial Cancer Research Fund Cancer Epidemiology Unit, University of Oxford, Oxford, UK; Office for National Statistics, 1 Drummond Gate, London, SW1V 2QQ, UK

**Keywords:** curability, person–years, survival, relative survival, routine data, trends

## Abstract

Because routinely collected survival data for cancer patients in England and Wales do not typically specify cause of death, conventional estimates of survival in cancer patients based on such data are a measure of their mortality from all causes rather than their mortality due to cancer. As a result, trends in survival over time are difficult to interpret because changes in overall survival may well reflect changes in the risk of death from other causes, rather than from the cancer of interest. One way of overcoming this problem is to use some form of ‘relative survival’ defined as a measure of survival corrected for the effect of other independent causes of death. Since this concept was first introduced, various methods for calculating relative survival have been proposed and this had led to some confusion as to the most appropriate choice of estimate. This paper aims to provide an introduction to the concept of relative survival and reviews some of the suggested methods of estimation. In addition, a particularly simple, but robust approach, is highlighted based on expected and observed mortality. This method is illustrated using preliminary data from the Office for National Statistics on cancer survival in patients born after 1939 and diagnosed with cancer during 1972–84. The examples presented, although limited to analyses on a small number of selected sites, highlight some encouraging trends in survival in people aged under 35 diagnosed with leukaemia, Hodgkin's disease and testicular cancer during this period. © 1999 Cancer Research Campaign


*British Journal of Cancer* (1999) **79**(1), 18–22
© 1999 Cancer Research Campaign

### Estimating relative survival among people registered with cancer in England and Wales

**GK Reeves[1], V Beral[1], D Bull[1] and M Quinn[2]**

[1]Imperial Cancer Research Fund Cancer Epidemiology Unit, University of Oxford, Oxford, UK; [2]Office for National Statistics, 1 Drummond Gate, London SW1V 2QQ, UK

**Summary** Because routinely collected survival data for cancer patients in England and Wales do not typically specify cause of death, conventional estimates of survival in cancer patients based on such data are a measure of their mortality from all causes rather than their mortality due to cancer. As a result, trends in survival over time are difficult to interpret because changes in overall survival may well reflect changes in the risk of death from other causes, rather than from the cancer of interest. One way of overcoming this problem is to use some form of ‘relative survival’ defined as a measure of survival corrected for the effect of other independent causes of death. Since this concept was first introduced, various methods for calculating relative survival have been proposed and this had led to some confusion as to the most appropriate choice of estimate. This paper aims to provide an introduction to the concept of relative survival and reviews some of the suggested methods of estimation. In addition, a particularly simple, but robust approach, is highlighted based on expected and observed mortality. This method is illustrated using preliminary data from the Office for National Statistics on cancer survival in patients born after 1939 and diagnosed with cancer during 1972–84. The examples presented, although limited to analyses on a small number of selected sites, highlight some encouraging trends in survival in people aged under 35 diagnosed with leukaemia, Hodgkin’s disease and testicular cancer during this period.

**Keywords:** curability; person–years; survival; relative survival; routine data; trends

One approach to assessing progress against cancer is to examine changes in the survival of cancer patients over time. Survival data for patients registered with cancer are compiled centrally by the Office for National Statistics (ONS) from information collected by regionally based cancer registries, and these data provide the main source of routine survival statistics for England and Wales. Although the ONS data include information on time from diagnosis to death, the specific cause of death is not routinely collected and so conventional estimates of survival based on these data reflect death from all causes rather than just the cancer of interest. Corresponding trends in survival estimates are sometimes difficult to interpret because it may be unclear whether changes in survival over time among a given group of cancer patients are in fact due to changes in the risk of death from the cancer itself, or to changes in the risk of death from causes other than that cancer.

In situations like this, it is common to use some form of ‘relative survival rate’ (Ederer et al, 1961) which is usually defined as a measure of survival ‘corrected’ for the effect of other independent causes of death, commonly referred to as background mortality. Although this approach appears simple in principle, there has been considerable debate in the statistical literature as to the correct choice of estimate, and it has been shown that use of certain estimates can lead to spurious results for long-term relative survival (Hakulinen, 1977).

*Received 27 November 1997*
*Revised 7 May 1998*
*Accepted 12 May 1998*

*Correspondence to:* GK Reeves, ICRF Cancer Epidemiology Unit, Gibson Building, Radcliffe Infirmary, Oxford OX2 6HE, UK

This paper aims to provide a straightforward introduction to the concept of relative survival and reviews some of the suggested methods for estimation. In particular, a simple person–years approach is described that is based on directly interpretable measures such as observed and expected numbers of deaths. This approach is illustrated using preliminary data from ONS on cancer survival in patients born after 1939 who were diagnosed with cancer during the period 1972–84.

### Materials and methods

The concept of relative survival was devised to provide an objective measure of the proportion of patients dying from the direct or indirect consequence of a disease in a given population and, hence, a measure of survival corrected for the effect of other independent causes of death. The basic definition of the relative survival rate is given below.

Let $T^d$ denote the time to death assuming that the subject is only at risk of death from the disease of interest, $T^e$ the time to death assuming that the subject is only at risk of death from all other causes, and $T$=minimum($T^d$,$T^e$) the observed time to death, all measured from some suitable reference point such as date of diagnosis. Assuming that the cause of interest and all other causes are independent, the overall probability of surviving to time $t$ is

$$\Pr(T \geq t) = \Pr(T^d \geq t)\,\Pr(T^e \geq t),$$

hence,

$$S^r(t) = \Pr(T^d \geq t) = \frac{\Pr(T \geq t)}{\Pr(T^e \geq t)} = \frac{S(t)}{S^e(t)} \qquad (1)$$

**Table 1** 5-year relative survival rates for patients diagnosed with cancer 1972–84 by primary site

| Cancer (ICD code under: 8th revision, 9th revision) | Age at diagnosis (years) | Total number of registrations | Per cent 5-year relative survival rate (95% CI) for years of diagnosis | | | |
|---|---|---|---|---|---|---|
| | | | 1972–75 | 1976–78 | 1979–81 | 1982–84 |
| Breast cancer (174, 174) | 15–34 | 5893 | 57 (55–60) | 60 (57–62) | 61 (58–63) | 62 (59–64) |
| Testicular cancer (186, 186) | 15–34 | 5220 | 65 (62–67) | 73 (70–76) | 85 (83–87) | 89 (87–91) |
| Hodgkin's disease (201, 201) | 15–34 | 6300 | 76 (74–77) | 80 (78–82) | 82 (80–84) | 87 (85–89) |
| Acute lymphatic leukaemia | 0–14 | 3659 | 45 (42–48) | 52 (49–55) | 64 (60–67) | 74 (70–77) |
| (204, 204) | 15–34 | 1044 | 22 (18–28) | 30 (25–36) | 34 (28–40) | 44 (38–50) |
| Acute myeloid leukaemia | 0–14 | 598 | 9 (6–13) | 18 (12–25) | 19 (13–26) | 34 (25–42) |
| (205, 205) | 15–34 | 1324 | 6 (4–8) | 14 (10–18) | 20 (16–25) | 31 (26–36) |

where $S^e(t)$ denotes the probability of survival to time $t$ if at risk from all other diseases, and $S(t)$ is the observed probability of survival to time $t$. The ratio $S^r(t)$ is termed the relative survival rate and can be viewed as the probability of survival to time $t$ with the disease of interest in the absence of the risk of death from other causes. Unlike crude survival, which would be expected to decline with increasing time, a levelling off in the relative survival rate after a given time, $t$, can occur if the individual is no longer at risk of death from the disease of interest. Thus, an apparent flattening of the cumulative relative survival curve is often taken to indicate that individuals who survive a given length of time are effectively 'cured' of the disease.

In general, $S(t)$ is estimated by the observed life–table estimate, based on the study cohort. The term $S^e(t)$ is substituted by some estimate of the expected probability of survival to time $t$, from all causes other than the disease of interest, for a group similar to that under study. Most of the discussion of the relative survival rate has centred on the choice of estimate for $S^e(t)$. The simplest approach, adopted by Ederer et al (1961), is to calculate the expected $t$-year survival probability for each individual alive at the beginning of the follow-up, based on relevant available life–tables, and take $S^e(t)$ to be the average of these values; in other words:

$$S^e(t) = \frac{1}{N} \sum_j S^e{}_j(t), \tag{2}$$

where $N$ is the total number of individuals in the study cohort. This estimate of $S^e(t)$ depends only on the composition of the cohort at the beginning of the study and takes no account of the subsequent withdrawal pattern.

The main problem with the estimate in equation 1, which was originally identified by Hakulinen (1977), becomes apparent when long-term relative survival rates are considered. Suppose that we are interested in the overall relative survival rate for a heterogeneous cohort; using equations 1 and 2, this can be expressed as the following weighted average

$$S^r(t) = \frac{\Sigma_j n_j S_j(t)}{\Sigma_j n_j S^e_j(t)} = \frac{\Sigma_j n_j S^r_j(t)\, S^e_j(t)}{\Sigma_j n_j S^e_j(t)} = \Sigma_j w_j S^r_j(t), \tag{3}$$

where $j$ is taken to denote age group or any other prognostic factor, $n_j$ is the number of individuals in the $j$th group at the start of the study and

$$w_j = \frac{n_j S^e_j(t)}{\Sigma_j n_j S^e_j(t)}, \; j = 1, \ldots, m.$$

As $t$ becomes large, $w_j$ tends to unity for the group with the best 'expected survival', $S^e_j(t)$, and to zero for all other groups. Thus, as the time period of interest increases, the overall relative survival estimate will tend to that of the group with the best expected survival, usually the youngest group. If one plots the relative survival rate (equation 3) for a heterogeneous population as a function of time, it sometimes appears as if relative survival starts to increase after a long period of time. Many people have attributed this to the possibility that improved medical care of cancer patients prevents them dying of causes other than their primary disease, but it may well be a spurious effect of using the form equation 2 as an estimate of $S^e(t)$ (Hakulinen, 1977).

If the expected survival of the study group was in itself of interest, equation 2 would be the correct estimate because it is based entirely on the composition of the study group at time 0 and is unaffected by the subsequent withdrawal pattern. However, the other component in equation 1, the observed survival $S(t)$, is heavily dependent on the pattern of withdrawal. The estimate of $S^e(t)$ should also, therefore, take into account the changing composition of the cohort by, for example, replacing $S^e(t)$ with a life–table estimate of expected survival as proposed by Ederer and Heise (1959). A simpler alternative approach, however, is to use the notion of excess death rates based on observed and expected mortality as suggested, for example, by Pocock et al (1982). In their paper, Pocock et al use excess deaths rates to make valid comparisons of survival between groups with potentially different background mortality rates. In what follows, we assume that interest lies specifically in estimating relative survival and, therefore, show how to translate excess or net mortality into corresponding relative survival estimates and derive corresponding approximate confidence intervals.

For each year (or other suitable time interval) after diagnosis, let $O$ denote the observed number of deaths in the study cohort, $E$ the expected number of deaths from causes other than the disease of interest (based on appropriate regional rates and taking into account calendar period, age and/or other relevant factors), and $Y$ the number of person–years contributed during that interval. The 'net mortality rate', that is the difference between the observed

© Cancer Research Campaign 1999

mortality rate and that which would be expected from all other causes, in the $i$th year after diagnosis is simply

$$\lambda_i = \frac{(O_i - E_i)}{Y_i}.$$

This net death rate is taken to represent the death rate due to the cause of interest in the $i$th year. If patients who survive to time $t$ are no longer at any excess risk of death relative to the general population then the net mortality rate at time $t$ should be 0 and this can be used as a statistical test of 'curability'. The cumulative $t$-year net mortality rate can be expressed as

$$\Lambda(t) = \Sigma^t_1 \lambda_i,$$

and, hence, the $t$-year relative survival rate is given by

$$S^r(t) = e^{-\Lambda(t)}. \quad (4)$$

Approximate confidence intervals for the relative survival estimate, $S^r(t)$, can be derived by assuming a normal distribution for log {–log $S^r(t)$}, with estimated variance

$$\sigma^2 = \frac{\Sigma^t_1 \, O_i / Y_i^2}{\{\Sigma^t_1 (O_i - E_i)/Y_i\}^2}.$$

Thus, an approximate 95% confidence interval for $S^r(t)$ is given by

$$S^r(t)^{\exp(\pm 1.96\sigma)}$$

Person–years for each time interval and the corresponding expected number of deaths can be obtained using any of the standard algorithms, for example PERSON–YEARS (Coleman et al, 1986), and the remaining calculations are easily programmed.

Several other approaches to the estimation of relative survival have been proposed (Hakulinen, 1982, 1985), but these are generally more complex than those described here. The person–years approach described above can be thought of as a special case of a more generalized modelling approach proposed by Esteve et al (1990) in which the net mortality rate is allowed to depend on any number of explanatory variables. Such a modelling approach is clearly more appropriate when primary interest lies in assessing which of many potential factors are important in determining mortality from the cause of interest. In some situations, it may be of interest to compare survival in a group of patients with their expected survival given their observed values of certain relevant covariates. In this case, Thomsen et al (1991) consider a continuous time analogue of relative survival in which the component representing expected survival takes into account historical information about the relationship between survival and the covariates of interest.

### RESULTS

### Application of the method to the analysis of survival of young patients diagnosed with cancer in England and Wales during 1972–84

The data used here are a subset of the national data on patients with cancer, compiled by ONS, and are unique in that the vital status of every patient registered after 1971 has been specially checked against the National Health Service Central Register. This subset includes data for only a few cancer sites and is limited to people born after 1939. The examples in this paper are, therefore, limited to results of analyses on a small number of selected cancers in relatively young patients, aged under 35 at diagnosis, and diagnosed between 1972 and 1984. Recent redevelopment of the computer system at ONS, however, means that vital status can now effectively be confirmed for all individuals registered with cancer in England and Wales from 1971–90, and this initial report will, therefore, be followed by an analysis of the entire ONS database for this period.

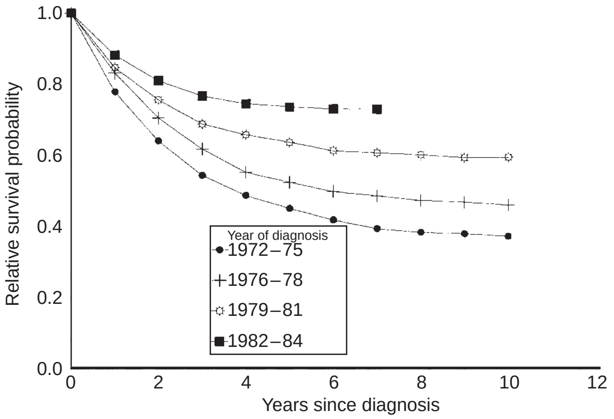

**Figure 1** Relative survival with acute lymphatic leukaemia in patients aged 0–14 diagnosed 1972–84

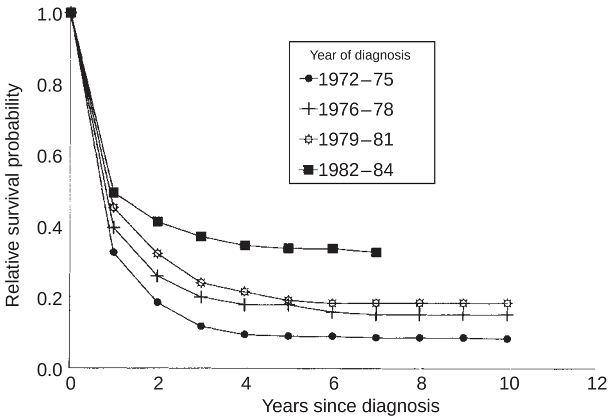

**Figure 2** Relative survival with acute myeloid leukaemia in patients aged 0–14 diagnosed 1972–84

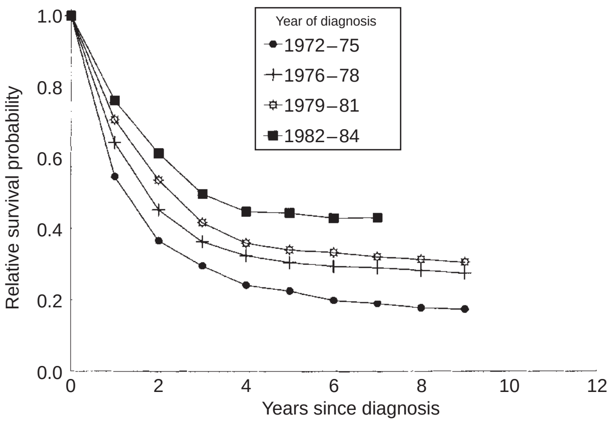

**Figure 3** Relative survival with acute lymphatic leukaemia in patients aged 15–34 diagnosed 1972–84

© Cancer Research Campaign 1999

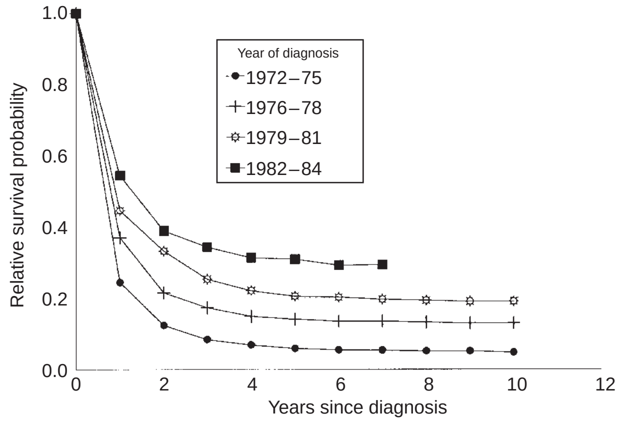

**Figure 4** Relative survival with acute myeloid leukaemia in patients aged 15–34 diagnosed 1972–84

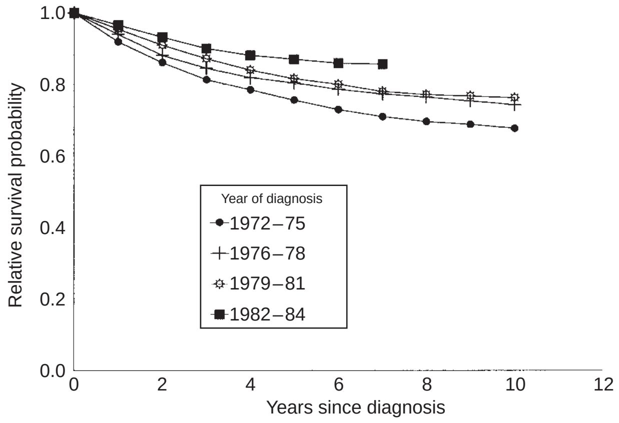

**Figure 6** Relative survival with Hodgkin's disease in patients aged 15–34 diagnosed 1972–84

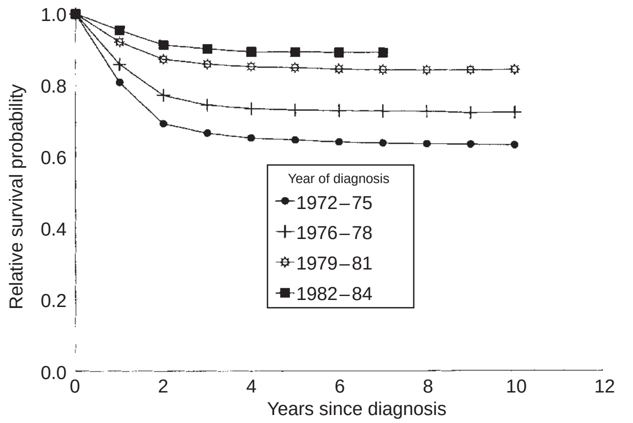

**Figure 5** Relative survival with testicular cancer in patients aged 15–34 diagnosed 1972–84

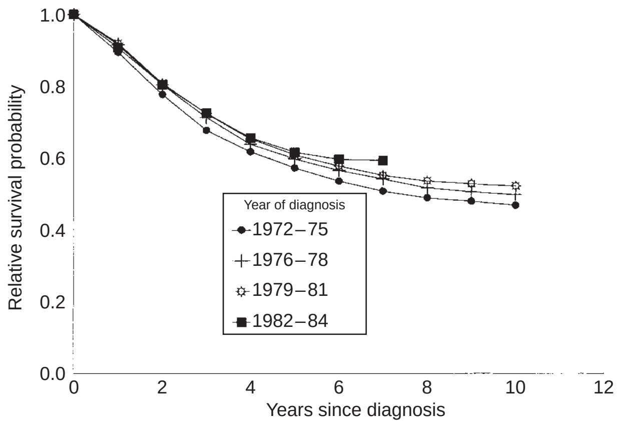

**Figure 7** Relative survival with breast cancer in patients aged 15–34 diagnosed 1972–84

As outlined in Materials and methods, time since diagnosis was divided up into yearly intervals; for each of these intervals, person–years and expected numbers of deaths were calculated using published death rates for England and Wales from all causes except that under study. All calculations were carried out using the PERSON–YEARS program (Coleman et al, 1986). For the purposes of these analyses, follow-up was censored at 1 January 1990 and cancers which were registered at the time of death were excluded. Results are shown separately for five selected sites. For each cancer, a plot of the cumulative relative survival rate (equation 4) was plotted against time since diagnosis. Because we are particularly interested in trends in survival over calendar period, relative survival curves are calculated separately for individuals diagnosed in periods 1972–75, 1976–78, 1979–81 and 1982–84. Figures 1–7 show plots of the cumulative relative survival up to 10 years after diagnosis for selected primary sites and age groups according to calendar period of diagnosis. Table 1 summarizes these results and gives the total number of registrations available for analysis in each case.

The results show that for some, but not all, of these cancers, survival for individuals aged 15–34 at diagnosis has improved substantially between 1972 and 1984. The most dramatic increases in survival shown here are for leukaemia. Because leukaemia is the most common childhood cancer, survival with acute lymphatic leukaemia (ALL) and with acute myeloid leukaemia (AML) is given both for children diagnosed under the age of 15 (Figures 1 and 2) and for adults aged 15–34 at diagnosis (Figures 3 and 4). The results for childhood leukaemia show that 5-year relative survival rates with AML increased from 9% in 1972–75 to 34% in 1982–84, whereas the rates for ALL increased from 45% to 74% over the same period. The results for adult leukaemias also show marked improvements in survival. In particular, 5-year relative survival rates for AML diagnosed at ages 15–34 rose from 6% in 1972–75 to 31% in 1982–84, whereas figures for ALL showed an increase from 22% to 44% over the same period. The results for testicular cancer (Figure 5) also show a gradual and significant increase in survival over this period, with 5-year relative survival rising from 65% in 1972–75 to 89% in 1982–84. Patients with Hodgkin's disease, aged 15–34 at diagnosis, showed some improvement in survival between 1972–84 (Figure 6) with 5-year relative survival increasing from 76% in 1972–75 to 87% in 1982–84. By comparison, survival with breast cancer among cancer patients aged 15–34 (Figure 7) showed relatively little change over this period.

*© Cancer Research Campaign 1999* 

### DISCUSSION

The main purpose of this paper is to illustrate a simple method of calculating relative survival based on routine data. Nevertheless, it highlights some interesting trends in the survival of young people diagnosed with certain cancers. Moreover, the patterns of survival observed are reassuringly in line with what is known about recent advances in the treatment of those cancers. For example, examination of the relative survival rates for testicular cancer by individual year between 1972 and 1984 suggest that the biggest increases occurred during the periods 1976–77 and 1979–80 (data not shown). This is likely to be due to the introduction of cisplatin-based chemotherapy, which was first used around 1976 before the commencement of widespread use in 1979 (Edmiston and Stewart, 1993). With regard to acute lymphatic leukaemia and acute myeloid leukaemia, the results shown here for children diagnosed under the age of 15 show similar patterns to those found by Stiller and Bunch (1990), who examined trends in survival with childhood cancers in England and Wales over the period 1971–85. The results also suggest that, to a certain extent, these improvements extend to young adults diagnosed at ages 15–34 and this finding is broadly in line with data on adolescent patients from Sweden (Adami et al, 1992).

One question which is often of interest in analyses of survival patterns in cancer patients is whether or not patients can ever be assumed to be effectively ‘cured’ of a particular cancer. One definition of curability, though not necessarily equivalent to a clinical definition, is when a patient assumes the same mortality rate as that of the general population. In such cases, the cumulative relative survival curve would be expected to level off after a given length of time. For some of the cancers shown here, such as leukaemia and testicular cancer, it is clear that this ultimately appears to be the case. For example, Figure 5 suggests that not only do as many as 89% of all testicular cancer patients diagnosed in 1982–84 survive beyond 5 years, but that those that do are no longer at any increased risk of death compared with the general population. For cancers such as breast cancer, in which there is no obvious levelling off in the cumulative relative survival rate before 10 years after diagnosis (Figure 7), the picture is less clear, and this has long been an area for discussion in the literature (Brinkley and Haybrittle, 1975; Duncan and Kerr, 1976; Langlands, 1995). In such cases, it is important to extend the estimates of cumulative relative survival to assess whether a certain proportion of patients ever reach a point at which they are no longer at an increased risk of death relative to the general population.

In this particular example, in which the data consist of relatively young patients, it is unlikely that the relative survival rates would have differed substantially from crude survival rates. There would, however, almost certainly be important differences if assessing trends in survival among older patients among whom a substantial proportion of the observed deaths would be likely to be due to other causes. Furthermore, although we have introduced the notion of relative survival in the context of analyses in which the cause of death is unavailable, there are arguments for applying this method to data in which cause of death can be ascertained from death certification data. Several authors have commented on the fact that even when cause of death has been routinely recorded, it is often difficult to determine whether a patient’s death was due directly or indirectly to their cancer and in those cases relative survival may provide a more objective means of removing the effect of mortality from other causes (Berkson and Gage, 1950).

In summary, this report discusses some of the issues surrounding the analysis of trends in survival based on routinely collected cancer registration data, and illustrates a relatively simple but robust method of calculating an estimate of relative survival which is suitable for use with large data sets. The examples presented here also highlight some very promising trends in the survival of young adults with certain cancers, particularly those with leukaemia and testicular cancer.

### REFERENCES

Adami HO, Glimelius B, Sparen P, Holmberg L, Krusemo UB and Ponten J (1992) Trends in childhood and adolescent cancer survival in Sweden 1960 through 1984. *Acta Oncol* **31**: 1–10

Berkson J and Gage RP (1950) Calculation of survival rates for cancer. *Proc Staff Meetings Mayo Clin* **25**: 270–286

Brinkley D and Haybittle JL (1975) The curability of breast cancer. *Lancet* **2**: 95–97

Coleman M, Douglas A, Hermon C and Peto J (1986) Cohort study analysis with a FORTRAN computer program. *Int J Epidemiol* **15**: 134–137

Duncan W and Kerr GR (1976). The curability of breast cancer. *Br Med J* **2**: 781–783

Ederer F and Heise H (1959). *Instructions to IBM 650 Programmers in Processing Survival Computations*. Methodological note no. 10. Bathseda, MD: End Results Evaluation Section, National Cancer Institute

Ederer F, Axtell LM and Cutler SJ (1961). The relative survival rate: a statistical methodology. *Natl Cancer Inst Monogr* **6**: 101–121

Edmiston KL and Stewart FM (1993). Germ cell testicular cancer: advances in treatment. *Forum Trends Exp Clin Med* **6**: 574–583

Esteve J, Benhamou E, Croasdale M and Raymond L (1990) Relative survival and the estimation of net survival: elements for further discussion. *Stat Med* **9**: 529–538

Hakulinen T (1977) On long-term relative survival rates. *J Chronic Dis* **30**: 431–443

Hakulinen T (1982) Cancer survival corrected for heterogeneity in patient withdrawal. *Biometrics* **38**: 933–942

Hakulinen T (1985) A computer program package for relative survival analysis. *Comput Programs Biomed* **19**: 197–207

Langlands AO (1995) Prognostic factors and the curability of breast cancer. *Aust NZ J Surg* **65**: 630–633

Pocock SJ, Gore SM and Kerr GR (1982) Long term survival analysis: the curability of breast cancer. *Stat Med* **1**: 93–104

Stiller CA and Bunch KJ (1990) Trends in survival for childhood cancer in Britain diagnosed 1971–85. *Br J Cancer* **62**: 806–815

Thomsen BL, Keiding N and Altman DG (1991) A note on the calculation of expected survival illustrated by the survival of liver transplant patients. *Stat Med* **10**: 733–738

© Cancer Research Campaign 1999
